# MHESMMR: a multilevel model for predicting the regulation of miRNAs expression by small molecules

**DOI:** 10.1186/s12859-023-05629-x

**Published:** 2024-01-02

**Authors:** Yong-Jian Guan, Chang-Qing Yu, Li-Ping Li, Zhu-Hong You, Meng-meng Wei, Xin-Fei Wang, Chen Yang, Lu-Xiang Guo

**Affiliations:** 1 School of Information Engineering, Xijing University, Xi’an, China; 2 College of Grassland and Environment Sciences, Xinjiang Agricultural University, Urumqi, China; 3 School of Computer Science, North-Western Polytechnical University, Xi’an, China; 4 College of Agriculture and Forestry, Longdong University, Qingyang, China

**Keywords:** LINE, microRNA, Small molecule, Generally attributed multiplex heterogeneous network embedding, Machine learning

## Abstract

According to the expression of miRNA in pathological processes, miRNAs can be divided into oncogenes or tumor suppressors. Prediction of the regulation relations between miRNAs and small molecules (SMs) becomes a vital goal for miRNA-target therapy. But traditional biological approaches are laborious and expensive. Thus, there is an urgent need to develop a computational model. In this study, we proposed a computational model to predict whether the regulatory relationship between miRNAs and SMs is up-regulated or down-regulated. Specifically, we first use the Large-scale Information Network Embedding (LINE) algorithm to construct the node features from the self-similarity networks, then use the General Attributed Multiplex Heterogeneous Network Embedding (GATNE) algorithm to extract the topological information from the attribute network, and finally utilize the Light Gradient Boosting Machine (LightGBM) algorithm to predict the regulatory relationship between miRNAs and SMs. In the fivefold cross-validation experiment, the average accuracies of the proposed model on the SM2miR dataset reached 79.59% and 80.37% for up-regulation pairs and down-regulation pairs, respectively. In addition, we compared our model with another published model. Moreover, in the case study for 5-FU, 7 of 10 candidate miRNAs are confirmed by related literature. Therefore, we believe that our model can promote the research of miRNA-targeted therapy.

## Introduction

As an emerging biomarker for medical and diagnostics, microRNA (miRNA) is a small single-stranded endogenously-initiated non-coding RNA molecule [1]. Since Ambros et al*.* discovered the first miRNA *lin-4*, about 28,000 miRNA molecules have been found in animals, plants and some viruses [2, 3]. Previously, the genomic structure and subtypes of protein, such as transcription factors and epigenetic mediators, were regarded as the only regulators of gene expression. However, researchers reveal the critical role of miRNAs in post-transcriptional regulatory mechanisms. Mature miRNA can bind to the 3’-untranslated region end of target mRNA, which triggers a decrease in the expression level of specific DNA [4]. This also suggests that miRNA expression levels affect multiple cellular functions, such as embryonic development, regulating substance metabolism, mediating signal transduction, cell division and apoptosis [5, 6]. In the human body, over 60% of transcription is regulated by miRNAs [7]. Since each miRNA can regulate the expression of many genes, each miRNA can regulate multiple cellular signalling pathways at the same time [8].

Cell activity is inseparable from the post-transcriptional regulation of miRNA. Meanwhile, many research papers indicated that the dysregulation of miRNA is related to disease occurrence, most notably cancer. Whether over-expression of carcinogenic miRNAs (oncomiRs) or down-regulation of tumor suppressor miRNAs (TSmiRs) may cause malignant tumours [9, 10]. Thus, miRNAs can be regarded as a biomarker for diagnosis [11]. People conducted a kind of medical treatment strategy based on the miRNA, called miRNA-target therapeutics [12, 13]. Its main modality is to regulate the expression level of oncomiRs or TSmiRs through SM. Since the special tertiary structure of miRNA, SM can bind to miRNA with high affinity and specificity. For example, Naro et al*.* discovered the first SM inhibitor of miRNA for suppressing the expression of miR-21 by the luciferase-base screening of more than 300,000 small molecules [14]. Miravirsen, a kind of oligonucleotide-based miR-122 inhibitor, has entered clinical trials and is well tolerated in non-human primates, which greatly reduces the burden of HCV and liver cancer [15]. Chandrasekhar et al*.* identified that aza-Flavanones could be an inhibitor of miR-4644, which was helpful to arrest and eliminate human breast tumor cells [16]. Besides, for a long time, it is extensively supposed that only proteins can be used as drug targets. But in fact, only about 600 kinds of disease modification proteins can be targeted by drugs. miRNA-targeted drugs are an important supplement to the pharmaceutical industry [17, 18]. In summary, discovering the regulation relation between miRNAs and small molecules harbours major implications for advancing miRNA-target therapeutics and drug development.

So far, the methods for discovering miRNA-target SM drugs can be divided into three categories. The first category is the high-throughput screening approach which uses high-throughput screening techniques to identify SM inhibitors or activators of miRNAs. For example, Zhang et al*.* presented a method based on miRNA 3D structure to discover miRNA-target SM which can regulate miRNA activity [19]. They utilized MC-fold to obtain miRNA structure. Similar to using Auto Dock to calculate the affinities between binding sites and ligand, they computed RNA-compatible score of SMs by molecule docking-based high-throughput screening techniques. Another category of approaches considers the structure of RNA base sequence. The most famous case is the web server of Inforna developed by Disney *et at.*, which predicts the association between SM-miRNA through motif alignment on a large scale in the databases [20]. The third category of the method is based on fluorescence detection assays. Bose et al*.* proposed a new method for identifying SM targeting miRNA in vitro using a molecule beacon [21]. The oligonucleotide hybridization probes are labelled with a fluorophore and a quencher when the beacon binds to the target miRNA. These studies have been instrumental in developing novel miRNA targeting SM drugs and old SM drug repositioning. Anyways, detecting the regulation of miRNAs expression by SMs through biological experiments is time-consuming and labor-intensive because the Bio-data is diverse and voluminous. Therefore, researchers intensified studies into developing computational methods to predict the association between SMs and miRNAs, hoping to narrow down the candidate drug searching scope and accelerate the process of drug development.

In recent years, a series of diverse computational models have been proposed to predict the association between miRNAs and SMs [22]. These miRNA-SM association prediction methods can be divided into two categories. The first category is sequence similartiy-based methods. For example, Lv et al*.* constructed an integrated SM-miRNA association network that combines the miRNA self-similarity network, SM self-similarity network and the known SM to miRNA targeting relationship network [23]. And they performed the improved random walk with restart algorithm (RWR) on the integrated SM-miRNA network, which allowed the random walk to learn samples on the various layers of the network. Finally, they ranked miRNA by the relevance score to each SMs, thus screening for potential miRNA targeting SMs. Jiang et al*.* leveraged the functional similarity of gene expression profiles under drug treatment and miRNA perturbation for SM-miRNA association prediction [24]. Meng et al*.* proposed the predicting model RWNS based on a three layers network including miRNA, SMs and diseases [25]. They considered multiple functional similarities such as SM chemical structure similarity, disease phenotype-based similarity and miRNA targeted gene functional consistency-based similarity. The integrated multiple types of functional similarities were constructed in a three layers network and implemented the random walk algorithm on the network. Deepthi et al*.* conducted a method to predict the relation between SM drugs and miRNA via the convolutional neural network (CNN). The miRNA similarity network and the SM similarity network were used as the features of miRNA and SM. The principal component analysis was implemented to reduce the dimensions of features and the CNN model was trained to extract the high-order information. Finally, they used the support vector machines for identifying the potential relation between miRNAs and SMs. Besides, Guan et al*.* developed the SM-miRNA association prediction model called the GISMMA model with the graphlet interaction-based inference [26]. The graphlet interaction aimed at describing the complex relationship between the miRNA similarity network and the SM similarity network. By counting the number of 28 types of graphlet interaction isomers, the GISMMA model can yield the predicted score of the potential relation between miRNA and SM. The second category is heterogeneous network-based methods. Li et al*.* presented the SMiR-NBI model to find miRNAs that can be the potential biomarkers for anticancer drugs. They constructed the SMiR-NBI model by a network-based inference. Specifically, they first initialized the resource scores of miRNAs based on the SM-miRNA adjacent matrix. Then the resource of miRNA was averagely distributed among the SM drugs that were directly linked to that miRNA in the network. Similarly, the SM drugs redistributed the resources to adjacent miRNAs after they integrated the resource from adjacent miRNAs. The final resource score of each miRNA represents the probability that it can be used as the biomarker for a certain anti-cancer drug.Wang et al*.* presented an approach of a triple layer heterogeneous network (TLHNSMA) to predict the association between SMs and miRNAs [27]. They exploited the functional similarities and relationships of miRNAs, SMs and diseases to construct a triple layers network. Then they developed an interactive updating algorithm to propagate the information across the three layers heterogeneous network. Anyways, there are three major disadvantages of these methods. First, most of the previous methods can only predict whether the SM can interact with miRNA but ought not to predict the regulation relation of the SM to the miRNA. These methods are unable to satisfy drug development and target selection because miRNAs may function as oncomiRs or TSmiRs. Thus, the key to advancing the research progress of miRNA-targeted therapy is to identify the SM modulators that inhibit oncomiRs and activate TSmiRs. Second, since most methods rely on the functional similarity of miRNA and SMs, these methods are constrained by complex side information. Therefore, there is a urgent need of an efficient and accurate auxiliary tool for the prediction of the SM regulation with miRNA.

One of the challenges in predicting the association between miRNA and SM is to identify whether their regulatory relationship is up-regulated or down-regulated. To address this challenge, we were inspired by the successful application of the attributed multi-layer heterogeneous network for predictions of multi-typed associations between miRNAs and diseases [28]. In this study, for predicting the miRNA-SM regulation relation, we introduced the attributed multi-layer heterogeneous network containing miRNA self-similarity and SM self-similarity. And we proposed a novel multilevel model called MHESMMR. The multilevel mdoel is composed of attributed multi-layer heterogeneous network and networks embedding methods. In detail, our proposed model consists of three steps. First, we carry out the LINE algorithm on the miRNA self-similarity and SM self-similarity for generating node features and then utilizes these node features to construct the attributed multi-layer heterogeneous network of miRNAs and SMs. And then, the GATNE algorithm is used for learning the representation features from the attributed multi-layer heterogeneous network. Finally, we feed these features into the LightGBM classifier to identify the probable SM modulators. To evaluate the performance of the proposed model, we predict the SM2miR under fivefold cross-validation. Furthermore, we compared the proposed model with other node feature extraction methods and machine learning classifiers, and the experiment results prove that the proposed model is a robust and efficient auxiliary tool for screening SM modulators for miRNA.

## Materials and methods

### Dataset

In the experiment, we collected the data about the regulation relation between SMs and miRNAs to evaluate the performance of the proposed model from the latest version of the SM2miR database [29]. The SM2miR database is a manually curated database that collected numerous SM’s effects on miRNA expression validated by the previous literature. According to the expression patterns of miRNA, the SM2miR database was divided into two parts, up-regulated pairs and down-regulated pairs, which correspond to Dataset 1 and Dataset 2, respectively. After pre-processing steps, we obtained 541 miRNA, 831 SM drugs and 2377 miRNA-SM pairs. Among these, 1394 up-regulation pairs belong to Dataset 1 and 983 down-regulation pairs belong to Dataset 2. The known SM-miRNA regulation relation pairs were regarded as positive samples.

In general, we describe a bipartite heterogeneous network of SM-miRNA regulation relations in which SM drugs and miRNAs are represented by nodes, and the relationships between them are represented by edges. The imbalanced problems may introduce bias into the experiment results. Thus, the same number of positive samples should be selected from unlabelled samples to generate the negative samples. In theory, the unlabelled samples selected in this manner may involve some potential SM-miRNA relation pairs. To do so, we carry out a negative sample selecting method based on the sequence proximity as similarly used by Yu et al*.* for negative sampling [30]. In terms of SM drugs, we generate MACCS fingerprints from SMILES to represent the SM drug chemical structure by the “RDKit” python package [31, 32]. To measure the proximity between each SM drug, we calculate the value of Tanimoto coefficients, a quantitative way for sequence alignment, based on their MACCS fingerprint.

Then, the regulation relations between any SMs and any miRNAs was computed. For example, we suppose that the regulation relation between miRNA1 and SM1 is unknown but miRNA1 can be inhibited by SM2, SM3 and SM4. The regulation relations between miRNA1 and SM1 can be calculated as follow:

1 $$r_{SM1}^{miRNA1} = \frac{{\overline{s}_{SM2} + \overline{s}_{SM3} + \overline{s}_{SM4} }}{3} = \overline{s}$$

where $\overline{s}$ denotes the mean value of Tanimoto coefficients of SM1-SM2, SM1-SM3, and SM1-SM4. We computed all of the regulation relations for unlabelled SM-miRNA pairs in the same way. Only the pairs of regulation relations score less than 0.1 were selected as the negative samples. Finally, we selected 1394 negative samples for Dataset 1 and 983 negative samples for Datset2.

### Node attributes of heterogeneous network by graph embedding

Graph embedding methods allow distributed representation of network structure, which can be divided into three categories including node embedding, edge embedding and substructure embedding. The node representation maps the nodes to the embedding space and each node can be represented by a vector. By doing this, the node embedding data containing the topological information of the graph are very effective inputs relative to the machine learning model for downstream classification tasks.

The LINE is a graph embedding method based on neighbourhood similarity assumptions proposed by Tang et al*.* and it is suitable for a weighted network [33]. In a complex network, if two vertices are direct neighbours, they are considered to have first-order proximity. On the other hand, if there are multiple first-order proximity vertices between two nodes, they are considered to have second-order proximity. From these two aspects, the main idea of the LIEN algorithm can be divided into two parts.

First-order proximity is to describe the local similarity in the graph. And the LINE with first-order proximity can only be applied to the undirected graph. The joint probability $p_{1}$ between two vertices $v_{i}$ and $v_{j}$ on the edge $e(i,j)$ can be defined as:

2 $$p_{1} (v_{i} ,v_{j} ) = \frac{1}{{1 + \exp ( - \vec{u}_{i}^{T} \vec{u}_{j} )}}$$

where $\vec{u}_{i}$ and $\vec{u}_{j}$ are the low-dimensional the low-dimensional representation vectors of $v_{i}$ and $v_{j}$. It can describe the relationship between vertices from the perspective of embedding space. The distribution $p(*,*)$ over the space $V \times V$ is defined as Formula (2). And its empirical probability $\hat{p}_{1}$ can be defined as:

3 $$\hat{p}_{1} (i,j) = \frac{{w_{ij} }}{W}$$

4 $$W = \sum\limits_{(i,j) \in E} {w_{ij} }$$

where $w_{ij}$ denotes the weight of the edge between vertices $v_{i}$ and $v_{j}$, and $W$ denotes the sum of all weights of the edges. The goal of our optimization formula is to minimize the difference between $p_{1}$ and $\hat{p}_{1}$, so the objective function is defined as follows:

5 $$O_{1} = d(p1(*,*),\hat{p}1(*,*))$$

where $d()$ represents the function used to measure the difference between two kinds of distributions. And the Kullback–Leibler (KL) divergence can be introduced to the above formula to replace the $d(*,*)$. The final optimized formula is defined as:

6 $$O_{1} = - \sum\limits_{(i,j) \in E} {w_{ij} logp_{1} } (v_{i} ,v_{j} )$$

Thus, all of the vertices can be represented as $\{ \vec{u}_{i} \}_{{i = 1...|{\text{V}}|}}$ in the d-dimensional space by optimizing the objective function.

The LINE also considers the second-order proximity between vertices. And the LINE with second-order proximity can be applied on both directed and undirected graphs. For a directed edge $e(i,j)$, the probability that vertex $v_{i}$ and vertex $v_{j}$ are directly connected can be defined as:

7 $$p_{2} = (v_{{\text{j}}} |v_{i} ) = \frac{{\exp (\vec{u}_{j}^{^{\prime}T} \cdot \vec{u}_{i} )}}{{\sum\nolimits_{k = 1}^{|V|} {\exp (\vec{u}_{k}^{^{\prime}T} \cdot \vec{u}_{i} )} }}$$

where $|V|$ denotes the number of vertices in the graph. And the empirical distribution is defined as:

8 $$\hat{p}_{2} (v_{j} |v_{i} ) = \frac{{w_{ij} }}{{d_{i} }}$$

where $d_{i}$ denotes the out-degree of $v_{i}$ and $w_{ij}$ denotes the weight of the edge $e(i,j)$. In order to make the low-dimensional representation of the conditional distribution of context $p_{2} ( \cdot |v_{i} )$ as close as possible to the empirical distribution $\hat{p}_{2} ( \cdot |v_{i} )$, the objective function can be defined as:

9 $$O_{2} = \sum\limits_{i \in V} {\alpha_{i} d(\hat{p}_{2} (*,*),p_{2} (*,*))}$$

where $\alpha_{i}$ denotes the prestige of the vertex $v_{i}$ and set as the degree of the vertex $v_{i}$ in this study. As mentioned above, $d(*,*)$ is replaced by KL-divergence. Thus, the final optimization function is defined as:

10 $$O_{2} = \sum\limits_{(i,j) \in E} {w_{ij} logp_{2} (v_{j} |v_{i} )}$$

Finally, each vertex can be represented by a d-dimensional vector $\vec{u}_{i}$ by finding $\{ \vec{u}_{i} \}_{{i = 1...|{\text{V}}|}}$ after minimizing the objective function. We applied the LINE algorithm to the miRNA self-similarity network calculated by the Tanimoto Coefficient. After graph embedding, if the properties of the two miRNAs are very similar, the embedding vectors between them will also be very close. We also performed the same operation on the SM self-similar network.

### Attributed multiplex heterogeneous network embedding

**Fig. 1:**
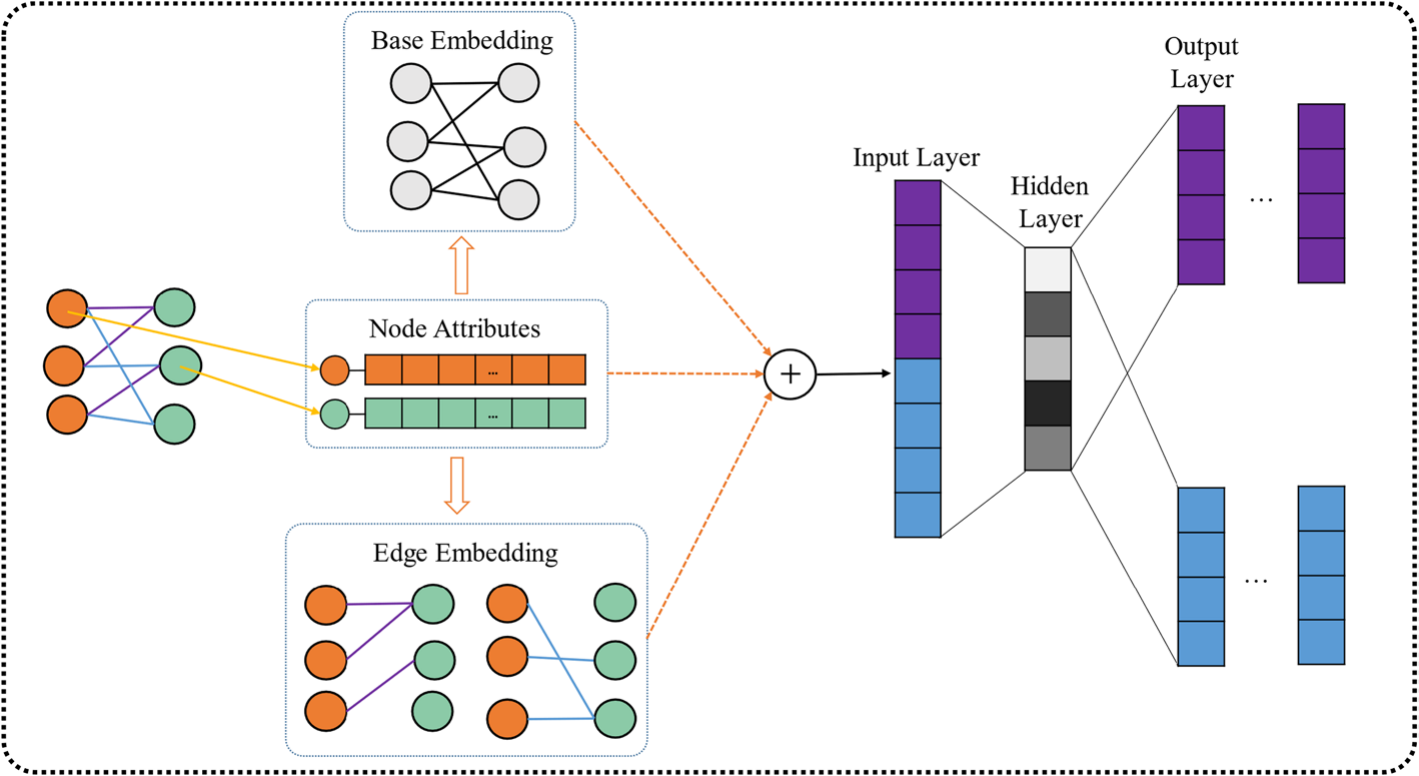
Illusion of GATNE in inductive mode

With the development of graph embedding, or network representation learning, exploring non-linear properties are critically important in extracting topological information from heterogeneous networks. There is an emerging graph embedding technology, called general attributed multiplex heterogeneous network embedding (GATEN). The GATNE algorithm aims to integrate the attribute features of the nodes and the multiple relationships between different types of nodes. Furthermore, it can project the information of nodes and non-linear relationships in the network into a relatively low-dimensional representation vector. Figure 1 shows the GATNE algorithm in inductive mode.

We assume that a relationship graph $G$ with a set of vertices $V = \{ v_{1} ,v_{2} ,...,v_{n} \}$, a set of edges $E = \{ e_{ij} |v_{i} ,v_{j} \in V\}$ and node attributed features $A = \{ x_{i} |v_{i} \in V\}$, that is ${\text{G}} = \{ V,E,A\}$. If the vertices and edges are of more than one type, $G$ is a multi-layer heterogeneous network that represents as $G_{r} = (V,E_{r} ,A)$ and $r$ denotes the types of relationships between two vertices. In general, the GATNE aggregates neighbour information and attributes information from the inductive context to the current vertices and generates feature vectors for each vertex at different layers. The GATNE is an inductive learning model with the combination of two parts: base embedding and edge embedding.

The base embedding of vertex $v_{i}$ is shared in different types of edges. The based embedding $b_{i}$ is calculated by a transform function defined as follow:

11 $$b_{i} = h_{z} \left( {x_{i} } \right)$$

where $h_{z}$ is a transformation function of attribute feature $x_{i}$ of vertex $v_{i}$ and the corresponding vertex type is represented by $z$.

In the edge embedding, the initial edge embedding $u_{i,r}^{(0)}$ for vertices is constructed by the transformation function with vertices attribute features ${\text{A}}$. as input. GraphSAGE is a graph neural network technology based on information aggregation [34]. The GATNE draws from the neighbour aggregator of the GraphSAGE to aggregate edge embedding vectors of vertex $v_{i}$ on layer $r$. The initial edge embedding and the mean aggregator function are as following:

12 $$u_{i,r}^{\left( 0 \right)} = g_{z,r} \left( {x_{i} } \right)$$

13 $$u_{i,r}^{(k)} = aggregator\left( {\{ u_{i,r}^{(k - 1)} ,\forall v_{j} \in N_{i,r} \} } \right)$$

where the transformation function of $z$ type vertex $v_{i}$ in relation $r$ is denoted as $g_{z,r}$. $u_{i,r}^{(K)}$ denoted the *K*-th level edge embedding after aggregation and $N_{i,r}$ represent the neighbour of vertex $v_{i}$ in relation $r$. Then all edge embedding $u_{i,r}$ in relation $r$ of vertex $v_{i}$ are concatenated as $U_{i}$ with size *s*-by-*m*, where s represents the dimension of edge embeddings:

14 $$U_{i} = (u_{i,1} ,u_{i,2} ,...,u_{i,m} )$$

The self-attention mechanism is performed on the $U_{i}$ to calculate the coefficients $c_{i,r} \in R^{m}$ of linear combination of edge embedding in $U_{i}$ on relation type of $r$, the function is formula as:

15 $$c_{i,r} = softmax(w_{r}^{T} \tanh (W_{r} U_{i} ))^{T}$$

where $w_{r}$ and $W_{r}$ are the trainable parameters of relation type $r$ and trained by optimization framework.

In general, the embedding representation vector of miRNAs and SM molecules on relation type $r$ are computed by the jointly optimization function as follow:

16 $$v_{i,r} = b_{i} + \alpha_{r} M_{r}^{T} U_{i} a_{i,r} + \beta_{r} D_{Z}^{T} x_{i}$$

where $b_{i}$ is the based embedding of vertex $v_{i}$.$\alpha_{r}$ is the hyper-parameter indicating the proportion of edge embedding in the entire embedding. And $M_{r} \in R^{s \times d}$ is trainable transformation matrix.

In parameter optimization framework, the GATNE integrated base embedding and edge embedding by the random walk and skip gram model on the attributed multi-layer heterogeneous network [35, 36]. Except random walk, meta-path-based methods are also commonly used in research in the field of bioinformatics in recent years. Meta-paths can be used to mine similarities and influences among network nodes. Based on these meta-paths, the similarity or weight between different nodes can be calculated to obtain more accurate recommendation results. At the same time, new relationships can also be discovered through meta-paths to improve the diversity and innovation of prediction models [37, 38].The meta-path-based random walk is used to generate vertices sequences to learn embedding. In detail, we suppose a graph $G_{r} = (V,E_{r} ,A)$ and a meta-path scheme $T:V_{1} \to V_{2} \to ...V_{t} ... \to V_{l}$, where $l$ is the length of the meta-path scheme. And the transition probability of random walk is defined as:

17 $$p(v_{j} |v_{i} ,T) = \left\{ {\begin{array}{*{20}l} {\tfrac{1}{{|N_{i,r} \cap v_{{_{t + 1} }} |}}} \hfill & {(v_{i} ,v_{j} ) \in E_{r} ,v_{j} \in V_{t + 1} } \hfill \\ 0 \hfill & {(v_{i} ,v_{j} ) \in E_{r} ,v_{j} \notin V_{t + 1} } \hfill \\ 0 \hfill & {(v_{i} ,v_{j} ) \notin E_{r} } \hfill \\ \end{array} } \right.$$

where $v_{i} \in V_{t}$ and $N_{i,r}$ is the neighbourhood of vertices $v_{i}$ in relation type $r$.. The meta-path-based random walk aims at digging out the semantic relationship between two different types of vertices for integrating by the skip-gram model. Finally, the objective function is defined as:

18 $$\begin{aligned} & E = - \log P_{\theta } (\{ v_{j} |v_{j} \in C\} |v_{i} ) \\ & \quad \quad = - \log \sigma (c_{j}^{T} \cdot v_{i,r} ) - \mathop \sum \limits_{l = 1}^{L} E_{{vk\sim P_{t} (v)}} [\log \sigma ( - c_{k}^{T} \cdot v_{i,r} )] \\ \end{aligned}$$

where $C$ is the context of vertex $v_{i}$ in the path $P = (v_{1} ,...v_{l} )$ and $c_{k}$ is the embedding of vertex $v_{i}$. $\sigma$ represents the sigmoid function and $L$ is the number of negative samples equal to positive samples. Among $v_{k}$ is randomly drawn from the distribution $Pt(v)$ which defined on the set of corresponding vertices $v_{i}$.

### LightGBM

In this study, we introduce a maching learning method as the classifier. LightGBM is a type of machine learning algorithm based on Gradient Boosting Decision Tree (GBDT) [39]. It is an efficient and fast gradient boosting framework developed by Microsoft. The lightGBM algorithm contains two novel techniques, namely Gradient-based One-Side Sampling (GOSS) and the Exclusive Feature Bundling (EFB), which can handle a large number of data instances and a large number of data features without overfitting problem, respectively [40]. LightGBM uses a histogram-based decision tree algorithm to discretize continuous features into discrete histogram features, thereby reducing data storage space and computational complexity. LightGBM uses a growth strategy called leaf-wise. The leaf-wise growth strategy selects the current optimal leaf node for splitting each time, which can quickly find the direction in which the loss function decreases the fastest, thus speeding up the training of the model.

### MHESMMR

**Fig. 2:**
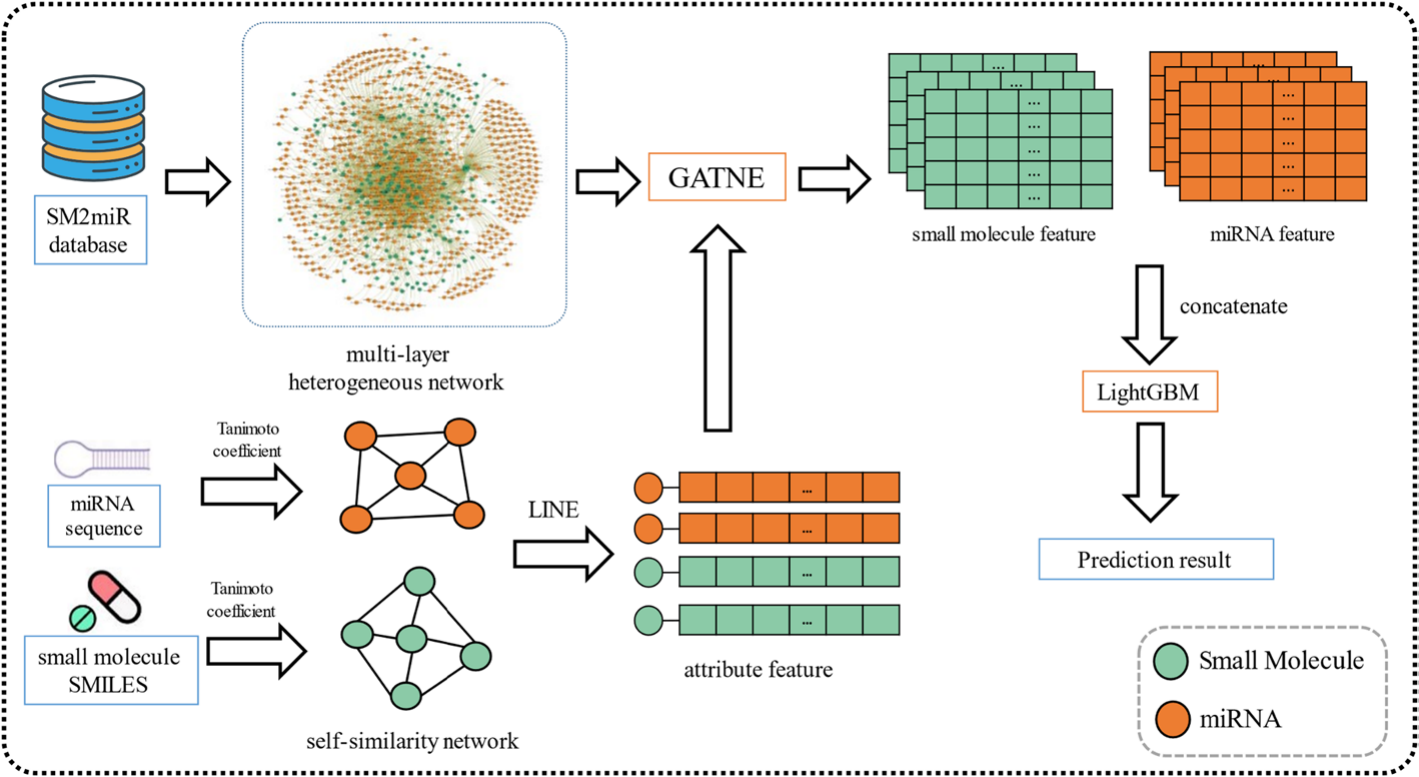
Framework of the MHESMMR model to predict miRNA-SM regulatory relations

In this work, owing to effective application of network embedding techniques in the bioinformatic field in the post-genomic era, we propose a novel computaional method named MHESMMR to predict multiple regulatory relations between miRNAs and SMs. MHESMMR can be describe in following five steps: (1) use the dataset to construct a multi-layer heterogeneous network, (2) construct the self-similarity networks of SM and miRNA by Tanimoto coeffcient, (3) generating node features by using LINE algorithm on the miRNA self-similarity and SM self-similarity network, (4) apply GATNE algorithm to aggregate the behavior information from the attributed multi-layer heterogeneous network for learning representation features(5) identify the probable SM modulators by the machine learning classifier, where the feature vectors of miRNA-SM are obtained by concatenating two representation features of corresponding miRNAs and SMs. The flowchart of the MHESMMR model is shown in Fig. 2.

## Experimental results and discussion

### Performance evaluation criterion

To validate the performance of the proposed model, we implemented a series of evaluation criteria. And fivefold cross-validation is adopted to ensure the rigor of the experiment. In detail, the positive samples and negative samples are equally divided into 5 folds. In each round of fivefold cross-validation, one of the folds is used as a testing sample set so that the prediction scores can be used using the proposed method. These prediction scores can reflect the possibility that an SM drug can regulate the expression of a miRNA. In our performance evaluation, if the positive sample in the test set has a high predictive score and the negative sample has a low predictive score, this indicates that the proposed model has good performance.

Moreover, we monitored accuracy (Acc.), sensitivity (Sen), specificity (Spec.) and Matthews Correlation Coefficient (MCC) to comprehensively evaluate the proposed model as follows:

19 $$Acc. = \frac{TN + TP}{{TN + TP + FN + FP}}$$

20 $$Sen. = \frac{TP}{{FP + FN}}$$

21 $$Spec. = \frac{TN}{{TN + FP}}$$

22 $$MCC = \frac{TP \times TN - FP \times FN}{{\sqrt {\left( {TP + FP} \right)\left( {TP + FN} \right)\left( {TN + FP} \right)\left( {TN + FN} \right)} }}$$

where TP is the number of positive samples that prediction score is higher than the threshold; FN is the number of positive samples that prediction score is lower than the threshold; FP is the number of negative samples that prediction score is higher than the threshold; TN is the number of negative samples that prediction score is lower the threshold, respectively. To show the results more intuitively, we drew the receiver operating characteristic (ROC) curves and precision-recall (PR) curves. The area under the ROC curve (AUC) and area under PR (AUPR) were also used for the evaluation of model performance [41, 42]. If the value of AUC is 0.5 that denotes a purely random prediction and 1 denotes a perfect prediction.

### Sensitivity analysis on parameters

**Fig. 3:**
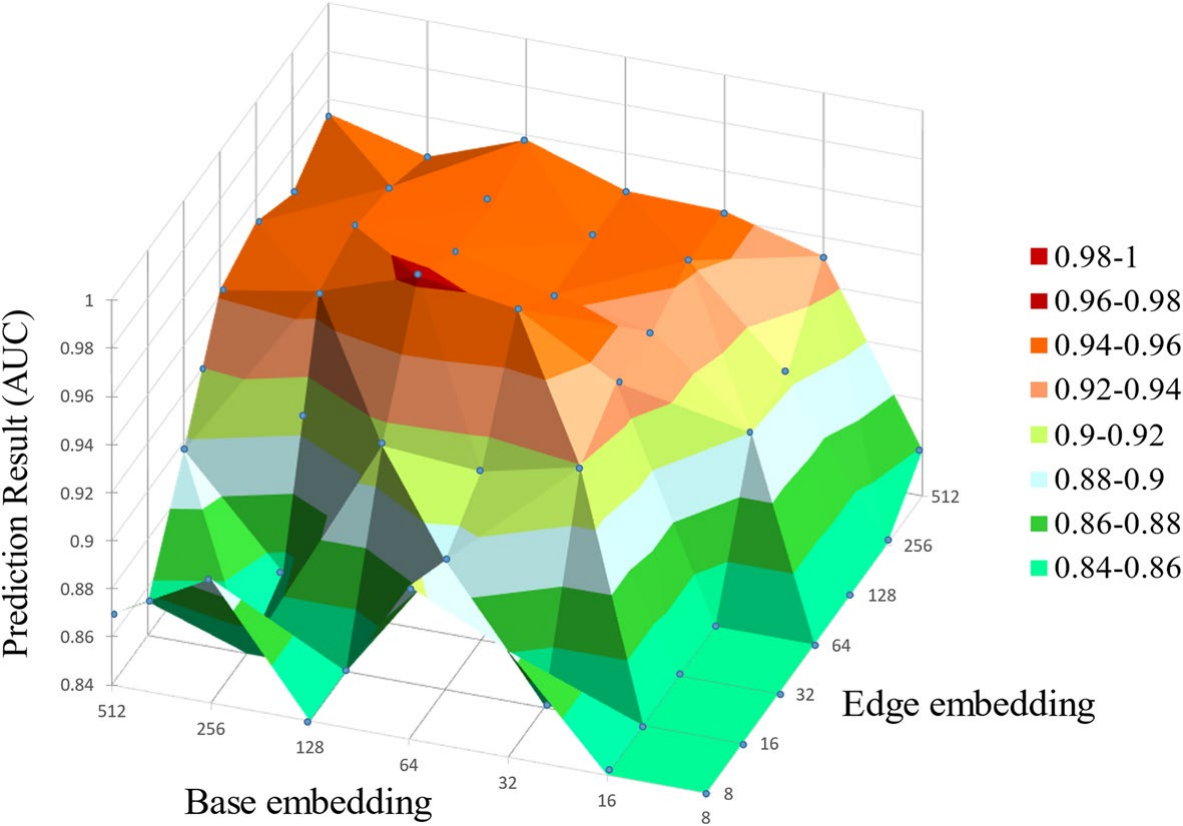
Sensitivity analysis on the dimension of base embedding and edge embedding

To obtain the best prediction performance, we performed the sensitivity analysis on the base embedding dimension and the edge embedding dimension. In this part, the sensitivity analysis was conducted on two hyper-parameters of the GATNE algorithm. Figure 3 illustrates the line chart of average AUC values, which was generated by the LightGBM classifier and influenced by the features dimension and the edge embedding dimension. It can be observed that when the base dimension is set to 128, the best results are obtained on the two data sets. The proposed model gets the best results when the edge embedding dimension of data set 1 and data set 2 is 64 and 32 respectively.

**Fig. 4:**
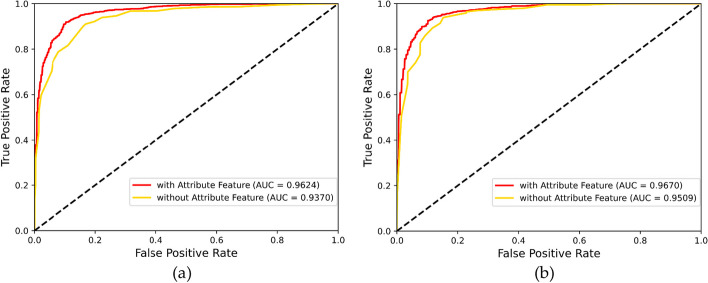
Difference of prediction performance using MHESMMR model with/without attribute feature input on Dataset1 (**a**) and Dataset2 (**b**)

In addition, an additional experiment was carried out to prove the effectiveness of the node attribute features generated by the self-similarity networks. Specifically, we removed the node attribute features and utilized the transductive mode to generate node features just relying on the network structure. As expected, without any node attribute feature, the MHESMMR model yielded average AUCs of 0.937 and 0.9509 on Dataset1 and Dataset2, which is lower than that obtained with attribute feature inputs. Figure 4 displays the ROC curve for this experiment. These results prove the combination of the attribute feature and the graph topology feature can improve the prediction performance.

### Assessment of prediction ability

**Fig. 5:**
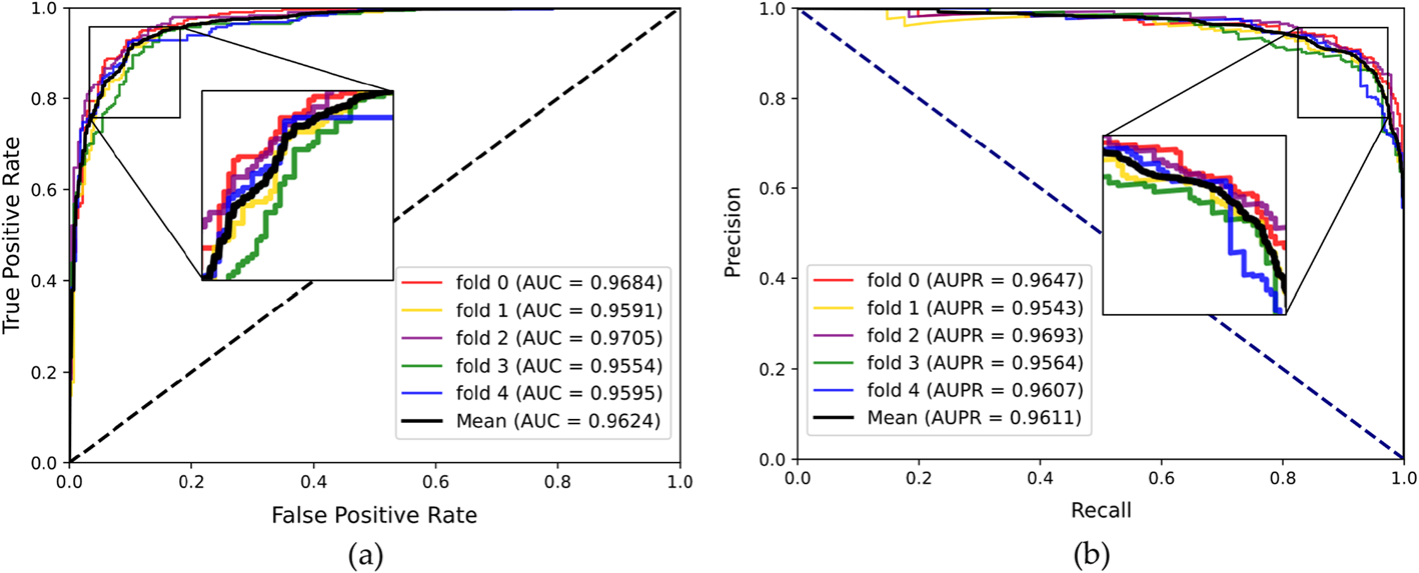
ROC curve (**a**) and PR curve (**b**) performed by MHESMMR on Dataset 1

**Fig. 6:**
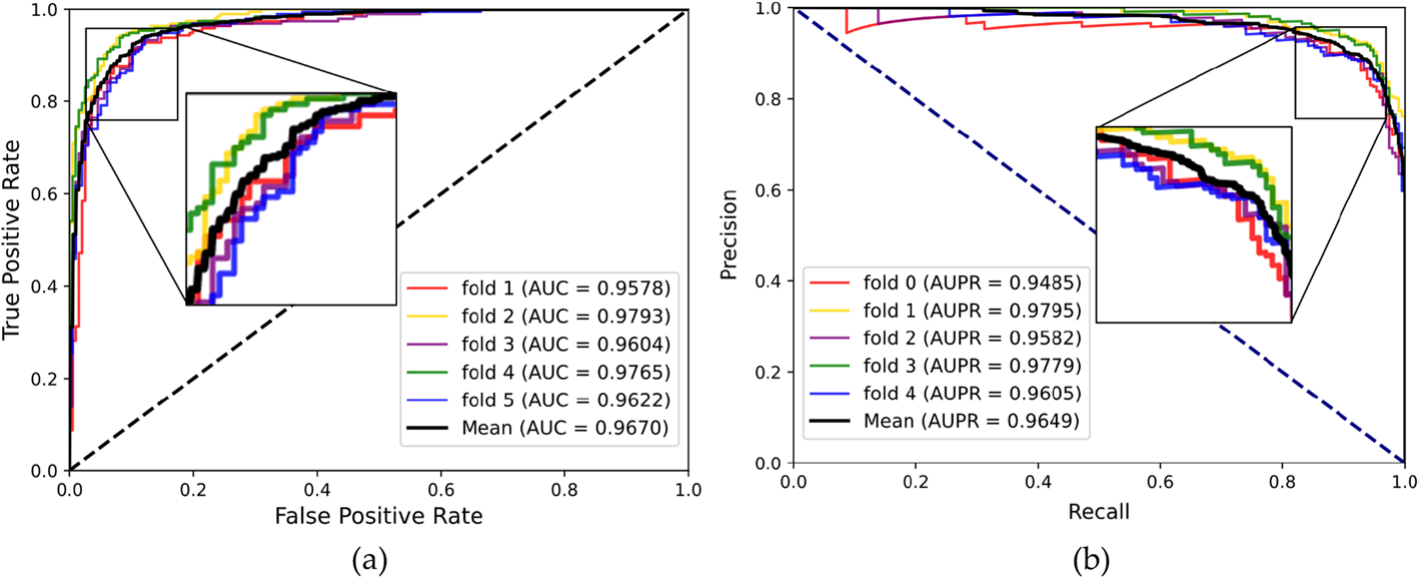
ROC curve (**a**) and PR curve (**b**) performed by MHESMMR on Dataset 2

To evaluate the prediction ability of the MHESMMR model while avoiding overfitting, we conducted fivefold cross-validation experiment on two datasets for our proposed model. To maintain consistency, all parameters of these experiments were consistent in this study. In Dataset1, we achieved the average results of Acc., Prec., Sen., MCC, AUC and AUPR of 90.55%, 92.73%, 89.25%, 82.10% 0.9624, 0.9607 and the standard deviations of 0.91%, 1.65%, 2.03%, 1.8%, 0.0065, 0.0050, respectively. In Dataset2, we obtained the average evaluation criteria of 90.97%, 92.74%, 85.28%, 79.98%, 0.9622, 0.9605 and the standard deviations of 1.51%, 1.76%, 3.25%, 2.94%, 0.0099, 0.1102, respectively. The results of the proposed model are summarized in the Table 1 and 2 when adopting the fivefold cross-validation on two datasets. The ROC and PR curves of the fivefold cross-validation experiment are shown in Figs. 5 and 6. All these results indicated a reliable predictive ability of our model.

**Table 1:** fivefold cross-validation performance for Dataset1

Fold	Acc. (%)	Sen. (%)	Spec. (%)	MCC (%)	AUC	AUPR
0	91.17	94.95	87.41	82.58	0.9684	0.9647
1	90.31	90.29	90.32	80.61	0.9591	0.9543
2	91.20	92.81	89.61	82.45	0.9705	0.9693
3	89.05	92.81	85.30	78.32	0.9554	0.9564
4	91.02	92.81	89.25	82.10	0.9595	0.9607
Average	90.55 ± 0.91	92.73 ± 1.65	88.38 ± 2.03	81.21 ± 1.80	0.9624 ± 0.0065	0.9611 ± 0.0050

**Table 2:** fivefold cross-validation performance for Dataset2

Fold	Acc. (%)	Sen. (%)	Spec. (%)	MCC (%)	AUC	AUPR
0	89.77	92.82	86.73	79.69	0.9578	0.9485
1	92.62	91.84	93.4	85.25	0.9793	0.9795
2	90.03	90.26	89.8	80.05	0.9604	0.9582
3	92.62	94.39	90.86	85.3	0.9765	0.9779
4	89.82	94.39	85.28	79.98	0.9622	0.9605
Average	90.97 ± 1.51	92.74 ± 1.76	89.21 ± 3.25	82.05 ± 2.94	0.9670 ± 0.0099	0.9649 ± 0.1102

### Ablation experiments

**Fig. 7:**
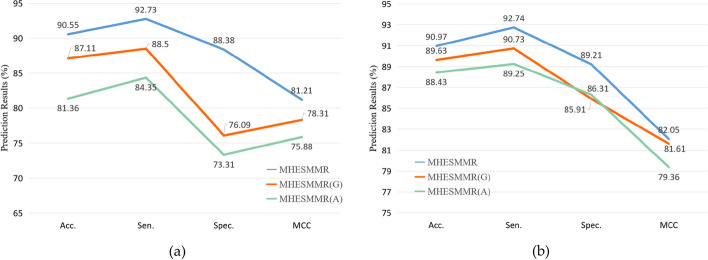
Ablation experiment result on Dataset1 and Dataset2

In MHESMMR model, the feature construction can be dived into two modules: node attribute feature construction and graph embedding feature construction. In ablation experiments, we verify which parts of the model contribute the most to the final performance. We constructed two prediction models using only one kinds of feature construction method. The first model only uses the GATNE algorithm to construct features, namely MHESMMR(G), which initial features of the attributed heterogeneous network are set to unit vectors. The second model is called MHESMMR(A), in which the extracted node features are directly input into the classifier to obtain prediction results. To ensure the fairness of the experiment, the same parameters and data set were used in all experiments. The experimental results were objectively recorded in Table 3. For the convenience of comparison, Fig. 7 was used to describe the comparison between the data in the ablation experiment and the original data. Figure 7 shows that the best prediction results can be achieved by combining the two models. Among them, MHESMMR(G) has a better prediction effect than MHESMMR(A), which proves that GATNE algorithm makes a greater contribution to the overall model.

**Table 3:** Ablation experiment result on Dataset1 and Dataset2

Model	Dataset	Acc. (%)	Sen. (%)	Spec. (%)	MCC (%)
MHESMMR	Dataset1	90.55 ± 0.91	92.73 ± 1.65	88.38 ± 2.03	81.21 ± 1.80
	Dataset2	90.97 ± 1.51	92.74 ± 1.76	89.21 ± 3.25	82.05 ± 2.94
MHESMMR(G)	Dataset1	87.11 ± 1.26	88.50 ± 2.39	76.09 ± 2.93	78.31 ± 2.41
	Dataset2	89.63 ± 3.22	90.73 ± 1.75	85.91 ± 3.71	81.61 ± 1.96
MHESMMR(A)	Dataset1	81.36 ± 1.54	84.35 ± 2.96	73.31 ± 3.69	75.88 ± 3.65
	Dataset2	88.43 ± 2.97	89.25 ± 3.01	86.31 ± 4.02	79.36 ± 2.45

### Performance by different classifiers

**Fig. 8:**
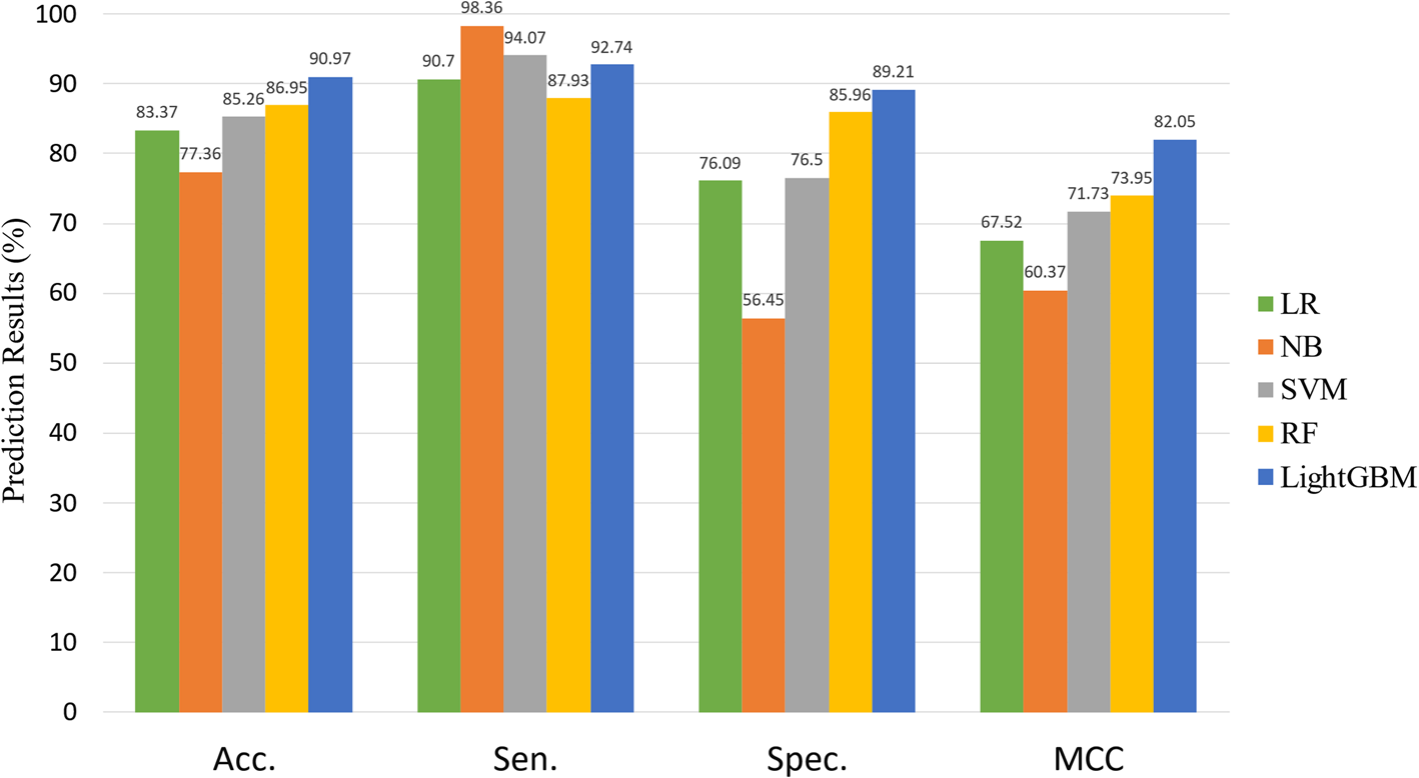
Experimental result of different classifiers on Dataset1

**Fig. 9:**
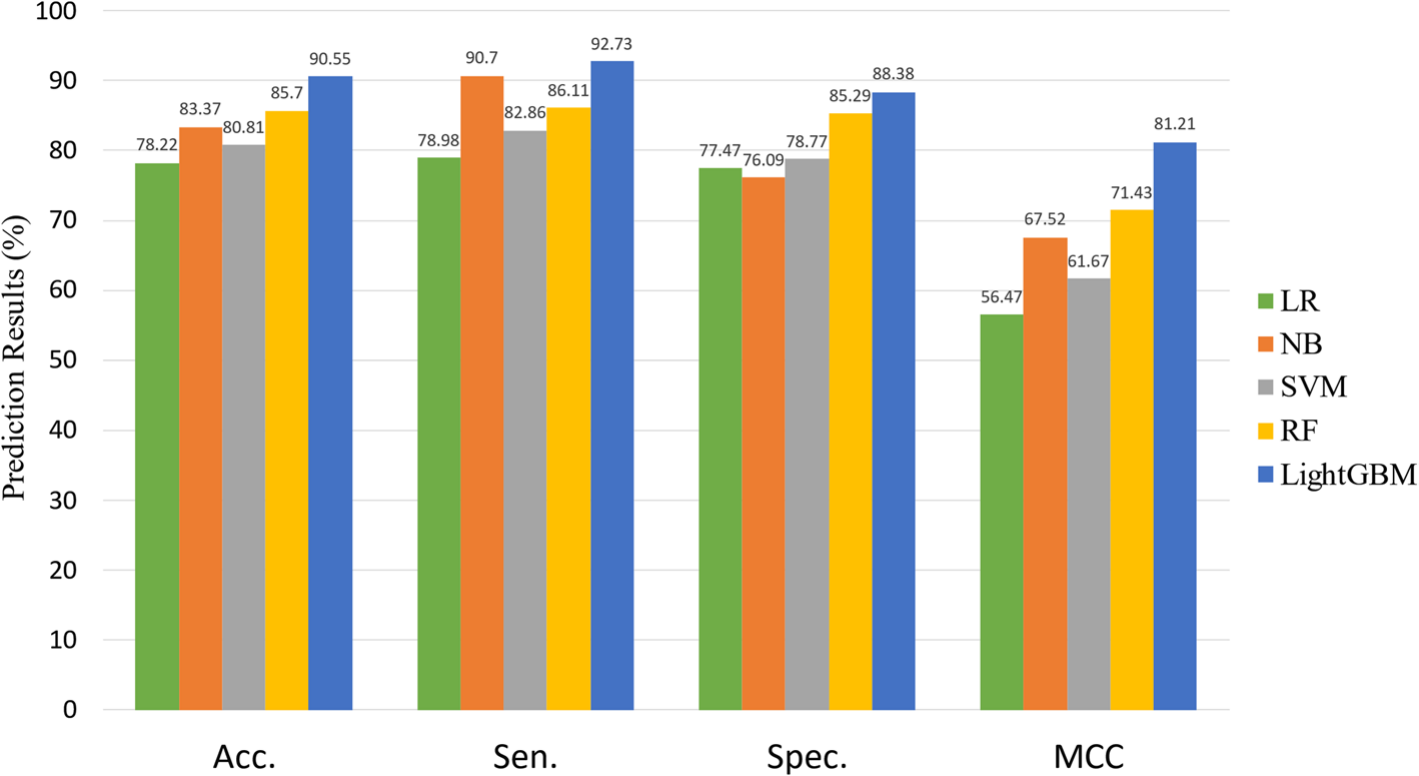
Experimental result of different classifiers on Dataset2

Machine learning algorithms are widely used in molecular interaction prediction models [43]. In order to prove the superiority of our classification strategy, we selected a number of classic algorithms commonly used in the field of bioinformatics to replace our classification method and compare the results.In the experiment, we used several popular machine learning algorithms to construct the prediction model including Logistic Regression (LR), Navi Bayes (NB), Support Vector Machines (SVM), Random Forest (RF) and LightGBM [44–48]. The performance of models based on Dataset1 from fivefold cross-validation is shown in Figs. 8 and 9.

When predicting miRNA-SM regulation relation for the Dataset1, we yielded average Acc., Sen., Spec., MCC, AUC and AUPR values of 90.55%, 92.73%,88.38%, 81.21%, 0.9594 and 0.9611S with corresponding standard deviations of 0.91, 1.65, 2.03, 1.80, 0.0065 and 0.0046, respectively. When predicting miRNA-SM regulation relation for the Dataset2, we yielded average Acc., Sen., Spec. and MCC values of 90.55%, 92.73%,88.38%, 81.21% with corresponding standard deviations of 0.91, 1.65, 2.03, 1.80, respectively. From these results, we can note that, among these five different prediction models, the LightGBM-based model achieved the highest Acc. on Dataset1 and Dataset2 of 90.55% and 90.97%. Moreover, the RF-based model yielded the second-highest Acc. Of 85.70% and 86.95%. Finally, the LightGBM model was selected for constructing the predicting model.

### Method comparison

**Fig. 10:**
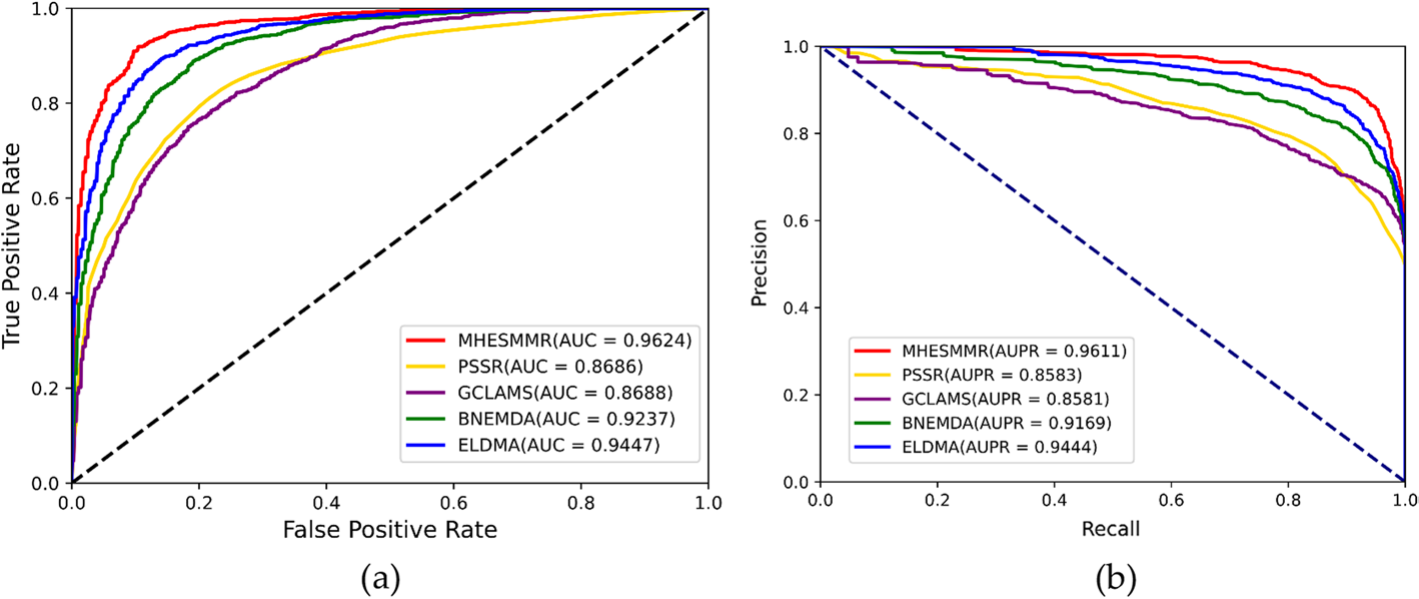
Prediction performance of models for SM-miRNA up-regulation pairs (Dataset1)

**Fig. 11:**
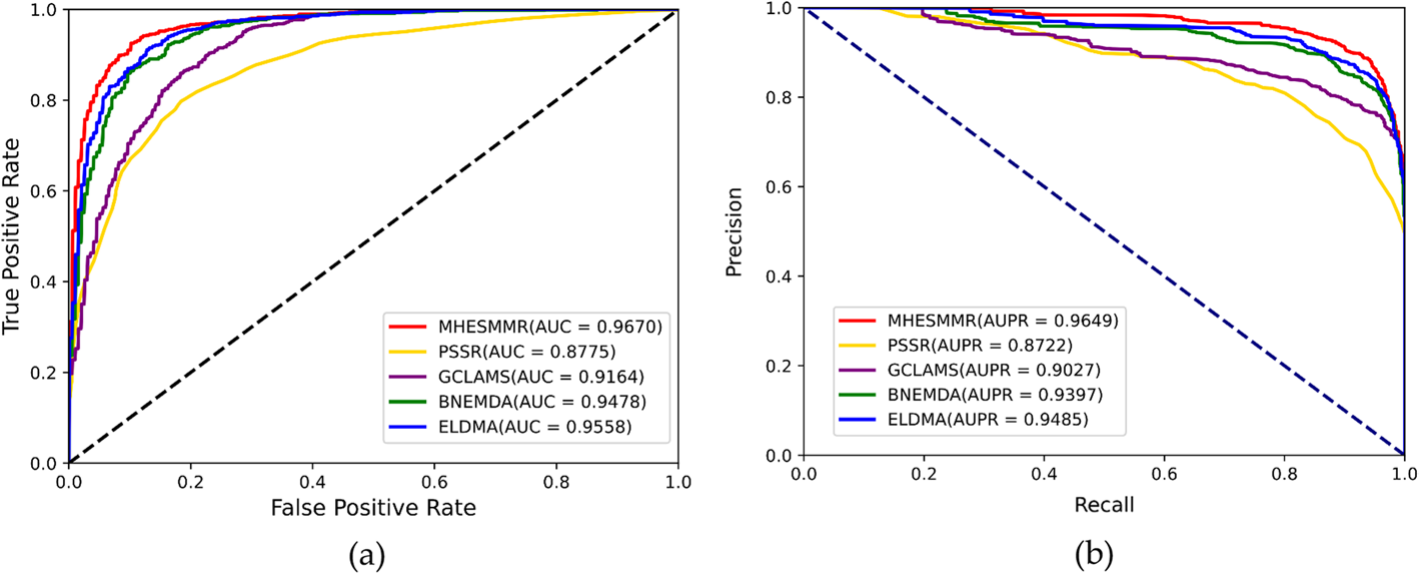
Prediction performance of models for SM-miRNA down-regulation pairs (Dataset2)

To my knowledge, the only computational model that can predict two types of regulatory relationship (up-regulated or down-regulated) between miRNAs and SMs is called PSRR [49]. To further demonstrate the predictive ability of the MHESMMR model, we compared it with the PSRR model on Dataset1 and Dataset2. The PSRR model constructs miRNA attribute features by 2-mer and 4-mer based on miRNA base sequences. And the166-dimensional MACCS fingerprints are calculated as the descriptors of SMs according to the SMILES of SMs. Finally, the PSRR model concatenates the two kinds of features and uses RF for classification prediction. In addition, we applied several previous models for predicting the interaction relationship between miRNA and small molecule drugs to our dataset, including BNEMDI, GCLAMS and ELDMA. BNEMDI used BiNE algorithm to extract tological feature in bipartite graph and predict miRNA-small molecule associaiton by DNN model. GCLAMS is a prediciton model based on heterogenous graph fusion neural netwok. ELDMA utilized the integrated pairwise similarities of small molecule and miRNA and convolutional neural network to extract intricate features. To show the results more intuitively, the result of the MHESMMR model and other models are compared in Figs. 10 and 11. According to Table 4, the AUC values of the MHESMMR model are 8.72% higher than the PSRR model in up-regulation pairs and 9.04% higher than the PSRR model in down-regulation pairs. The AUPR values of the MHESMMR model are 10.03% higher than the PSRR model in up-regulation pairs and 8.74% higher than the PSRR model in down-regulation pairs. These results clarified that the MHESMMR model, with the benefit from attributed multiplex heterogeneous network embedding, could be an accurate and efficient computational model for the prediction of the regulation of miRNAs expression by SM on a large scale.

**Table 4:** Comparison of experimental results of MHESMMR and PSSR

Model	Dataset	Acc. (%)	Sen. (%)	Spec. (%)	MCC (%)	AUC	AUPR
BNEMDI	Dataset1	84.69 ± 2.07	86.75 ± 2.97	82.64 ± 2.43	73.29 ± 2.09	0.9237 ± 0.0364	0.9169 ± 0.0145
	Dataset2	88.83 ± 0.55	90.29 ± 1.61	87.38 ± 2.31	77.74 ± 1.83	0.9478 ± 0.0045	0.9397 ± 0.0051
GCLAMS	Dataset1	80.37 ± 2.24	90.70 ± 2.28	76.09 ± 3.42	67.52 ± 4.37	0.8688 ± 0.2143	0.8581 ± 0.2325
	Dataset2	87.36 ± 2.52	86.36 ± 0.66	69.29 ± 3.81	69.37 ± 2.58	0.9164 ± 0.3548	0.9027 ± 0.1429
ELDMA	Dataset1	87.63 ± 2.08	88.84 ± 1.47	86.01 ± 3.46	74.91 ± 4.12	0.9447 ± 0.0015	0.9444 ± 0.1236
	Dataset2	88.93 ± 0.74	91.31 ± 1.68	86.57 ± 1.93	77.99 ± 1.45	0.9558 ± 0.3484	0.9485 ± 0.7123
PSRR	Dataset1	79.59 ± 1.67	78.47 ± 2.32	80.7 ± 2.43	59.22 ± 3.33	0.8689 ± 0.0134	0.8583 ± 0.0125
	Dataset2	80.37 ± 1.54	78.32 ± 1.94	82.4 ± 3.63	60.84 ± 3.21	0.8765 ± 0.0135	0.8722 ± 0.0133
MHESMMR	Dataset1	90.55 ± 0.91	92.73 ± 1.65	88.38 ± 2.03	81.21 ± 1.80	0.9594 ± 0.0065	0.9611 ± 0.0046
	Dataset2	90.97 ± 1.51	92.74 ± 1.76	89.21 ± 3.25	82.05 ± 2.94	0.9620 ± 0.0099	0.9649 ± 0.0062

### Case study

For validating the performance of MHESMMR model on predicting potentially the regulation of miRNAs expression by SM. We conducted a case study identifying miRNA targets of specific drugs. 5-FU (CID 3385) was selected as the designated drug for this case study. 5-FU is a kind of common chemotherapy drugs for cancer [50]. It can inhibit the proliferation of cancer cells by changing the metabolism of RNA and DNA to reduce the synthesis of specific proteins [51–54]. Therefore, we utilized the Dataset2 to construct the down-regulation pairs prediction model. We removed all of relation pairs between 5-FU and all of miRNAs in Dataset2 and then implement MHESMMR model based on rest SM-miRNA relation pairs. The prediction results are shown in Table 5. According to the Table 5, among the potential 5-fu-related miRNAs with the top 10 highest prediction scores, seven of them were proved by the PubMed literature to be inhibited by 5-FU.

**Table 5:** The top 10 predicted miRNAs interacted with the 5-FU

Rank	PubChem ID	miRNA	Possibility	Evidence
1	3385	hsa-miR-21-5p	0.9857	21,506,117
2	3385	hsa-miR-92a-3p	0.9733	19,956,872
3	3385	hsa-miR-16-5p	0.9660	Unconfirmed
4	3385	hsa-miR-15b-5p	0.9641	19,956,872
5	3385	hsa-miR-128-3p	0.9555	19,956,872
6	3385	hsa-miR-30a-5p	0.9542	21,506,117
7	3385	hsa-miR-155-5p	0.9518	Unconfirmed
8	3385	hsa-miR-191-5p	0.9385	19,956,872
9	3385	hsa-miR-210-3p	0.9317	17,702,597
10	3385	hsa-miR-107	0.9307	Unconfirmed

For instance, Shah et al. demonstrated 5-FU can down-regulate the expression of hsa-miR-21-5p by qRT-PCR. Hsa-miR-30a-5p can medita the effect of 5-FU on p53-mutatant cells which is resistant to 5-FU. MiRNAs affected by 5-FU can target important p53 regulatory genes. Zhou et al. had identified hsa-miR-92a-3p, hsa-miR-15b-5p, hsa-miR-191-5p and hsa-miR-128-3p as down-regulate in HCT-8 and HCT-116 colon cancer cells after exposure to 5-FU by microarray analysis [55]. Rossi et al. discovered down-regulation of hsa-miR-210-3p in 5-FU treated HC.21 cell lines.

## Conclusion

It is well known that the abnormal expression of miRNAs is an important role in various pathological processes. Through SM drugs, the oncomiRs can be down-regulated and the TSmiRs can be up-regulated. Therefore, an efficient miRNA drug regulatory relationship prediction model is needed. In this study, we developed an innovative computational method for the prediction of regulation relation between miRNA-SM based on graph embedding and machine learning named MHESMMR. It combines the LINE algorithm, the GATNE algorithm and the LightGBM method. And it shows the usefulness of non-linear relationships in identifying the potential miRNA-SM associations. For evaluating the performance of the proposed model, we divide the up-regulation pairs and down-regulation pairs in the SM2miR dataset into Dataset1 and Dataset2. And we performed tests on these two datasets under a fivefold cross-validation. The MHESMMR model yielded average accuracies of 79.59% and 80.37% for Dataset1 and Dataset2, respectively. In addition, we compare the proposed model with another existing model to verify the predictive ability of our model. We also compare the LightGBM method with other classical machine learning classifiers. The experimental results demonstrated that the MHESMMR model is a valuable tool to predict miRNA-SM regulation relations. In the future, we intend to search for more effective feature extraction methods and develop diverse feature descriptors to construct better prediction models.

## Data Availability

SM2miR [SM2miR (jianglab.cn)] is freely available to the public without registration or login requirements. The data and source code can be found at https: //github.com/Heath0/ MHESMMR.
